# 71. Paget's disease with rheumatoid arthritis: by chance not providence

**DOI:** 10.1093/rap/rky034.034

**Published:** 2018-09-20

**Authors:** Prasan Deep Rath, Swetal C Pandey, Shaloo Bhasin

**Affiliations:** Rheumatology, Department of Rheumatology, Delhi, INDIA

**Introduction:** Paget’s disease is a monostotic or polyostotic metabolic bone disease which is characterised by a focal bone remodeling, with increased bone resorption and new bone formation which is excessive and disorganized. Paget’s disease is rare in India, China, but its commonly seen in Western Europe, England and the United States. The study done on Western Indian population of Paget’s disease shows male preponderance with a mean age of onset at 62 years. In them it presented as pain, fracture and typical skeletal involvement. It is a non-familial in Indian population. Polyostotic disease involves commonly pelvis, skull, spine and femur. Rheumatoid arthritis is chronic debilitating with deforming disease with significant morbidity and mortality with prevalence of 0.75% in Indian population. Rheumatoid arthritis has been associated with Paget’s disease in 1% of cases, but there are not many recent studies or case reports to our knowledge.

**Case description:** A 65 year old south Asian female presented to the rheumatology outpatient clinic in 2015 with history of pain in both elbows. It was insidious in onset, gradually progressive. The patient described her pain well defined, persisting at rest, exacerbated both at night and by doing activity. She denied fever, night sweats, or weight loss. No history of pain in any other joints. After initial rheumatologic assessment, all laboratory parameters were normal except serum alkaline phosphatase which was two times as high as upper limit. Following which radiographs were taken, showing changes consistent with Paget’s disease: Cortical thickening, mixed areas of lucency and sclerosis, coarse trabecular markings and typical lytic and sclerotic changes. Radionuclide bone scan revealed markedly increased tracer uptake in distal left humerus, proximal left femur and right pelvic bone, suggestive of Paget’s disease. She was started treatment with bisphosphonate-zoledronic acid, vitamin D and calcium supplements, her symptoms gradually improved along with decline in alkaline phosphatase levels. She achieved remission in two years. She returned back to outpatient clinic with complaints of pain and swelling in small joints of hands after two months. The pain was worse in morning, with improvement with activity. She also had morning stiffness for more than two hours. On examination, swelling of bilateral metacarpophalangeal joint, wrist was present. Serum alkaline phosphatase levels were normal. Inflammatory markers were high. Rheumatoid factor was high (206 IU/ml) along with raised anti CCP (427.6 u/ML ). Ultrasound of wrist was done which showed synovial proliferation with doppler activity. She was started treatment with methotrexate 15 mg once weekly along with leflunomide and hydroxychloroquine. Following this her joint pain improved in three months.

**Discussion:** Paget’s disease of bone, rheumatologic diseases presenting as joint and bone pain are commonly seen in elderly people. Clinicians should be aware of the spectrum of clinical presentations of these diseases. All these disease can present with bone pain and joint pain .with overlapping symptoms . this makes them very hard to diagnose and treat these diseases .With bisphosphonates in Paget’s bone disease should eliminate bone pain, normalize serum total alkaline phosphatase, induce remission, heal radiographic osteolytic lesions, and prevent recurrence and complications. In a study by roy altman , Paget’s patients can present with arthritis . Most commonly 37% of 290 patients presented with back pain which was mostly related to spinal osteoarthritis while spondylitis was present only in 1% . 30% showed osteoarthritis of hip while 11% showed in knee . while Paget’s disease presenting with Rheumatoid arthritis was very rare seen in only 1% of patients. Two overlapping subsets of rheumatoid arthritis have been recognised among Paget’s and rheumatoid overlap - one with classical RA clinical picture as symmetrical polyarthritis , while other subset can present as PMR-like appearance, which is characterised by predominatly presented as non erosive joint and shoulder involvement and absence of rheumatoid factor. Our findings of high titres of RF, CRP, ESR, along with symmetric peripheral arthritis in elderly female patient have supported the diagnosis of RA along with USG of both wrist showing synovial proliferation with doppler activity . In such overlapping presentation, until one has thorough knowledge of spectrum of clinical diseases, other associated diseases can be easily missed and there can be delay in diagnosis.

**Key Learning Points:** All bone and joint pain in case of polyostotic Paget’s disease is not always due to Paget’s disease. Rheumatoid arthritis (1%) can rarely be seen in pagets while osteoarthritis related to Paget’s disease was most common, presenting in spine (37%) , hip (30%) and knee (11%). High index of clinical suspicion to rule out other rheumatological disorders coexisting in Paget’s is required.

**Disclosure: P. Rath:** None. **S.C. Pandey:** None. **S. Bhasin:** None.

